# EndoS Reduces the Pathogenicity of Anti-mCOL7 IgG through Reduced Binding of Immune Complexes to Neutrophils

**DOI:** 10.1371/journal.pone.0085317

**Published:** 2014-02-04

**Authors:** Xinhua Yu, Junfeng Zheng, Mattias Collin, Enno Schmidt, Detlef Zillikens, Frank Petersen

**Affiliations:** 1 Priority Area Asthma & Allergy, Research Center Borstel, Airway Research Center North (ARCN), Members of the German Center for Lung Research (DZL), Borstel, Germany; 2 Laboratory of Autoimmunity, The Medical College of Xiamen University, Xiamen University, Xiamen, China; 3 Division of Infection Medicine, Department of Clinical Science, Lund University, Lund, Sweden; 4 Department of Dermatology, University of Lübeck, Lübeck, Germany; Federal Institute for Vaccines and Biomedicines, Germany

## Abstract

Endo-β-N-acetylglucosaminidase (EndoS) has been shown to act as a potent pathogen-derived immunomodulatory molecule in autoimmune diseases. Here we investigated how EndoS treatment reduces the pathogenicity of rabbit anti-mCOL7 IgG using different experimental models of epidermolysis bullosa acquisita (EBA). Our results show that the EndoS treatment does not interfere with the binding of the antibody to the antigen but reduces immune complex (IC)-mediated neutrophil activation by impairing the binding of the IC to FcγR on neutrophils. On the basis of this newly identified EndoS-mediated mechanism we hope to develop new strategies in the treatment of the disease.

## Introduction

Endo-β-N-acetylglucosaminidase (EndoS) is a endoglycosidase secreted by *Streptococcus pyrogenes* that specifically hydrolyzes the β-1,4-di-N-acetylchitobiose core of the asparagine-linked glycan of human IgG [1]. It has evolved as a powerful tool of *Streptococcus pyrogenes* to combat human humoral defense system. EndoS has been shown to hydrolyze efficiently native IgG both *in vitro* and *in vivo*, which make it a good candidate for a pathogen-derived immunomodulatory molecule [2]. So far, the therapeutic efficacy has been shown in experimental models of many autoimmune diseases, including antibody-induced arthritis [3], immune-thrombocytopenic purpura [2], lupus-like disease [4], glomerulonephritis [5], and autoimmune hemolysis [6] This suggest that EndoS can be used as a powerful therapeutic molecule in the treatment of autoimmune diseases, especially those involving autoantibodies.

Recently, we have shown that treatment of EndoS results in the modulation of experimental epidermolysis bullosa acquisita (EBA) [7], an autoimmune skin blistering disease mediated by autoantibodies against type VII collagen [8]. The pathogenesis of this disorder involves the Fc portion of the autoantibodies as well as the complement system both mediating the activation of neutrophils which act as essential executer of tissue damage [9]–[11]. Several modeling systems of EBA have been established allowing the precise analysis of the disease during different phases. These models include the active immunization of mice with type VII collagen, a passive transfer of anti-mCOL7 IgG into animals, as well as an *ex vivo* model on cryosections and an *in vitro* neutrophil activation assay [10]–[13].

In this study, we applied the *ex vivo* model and the *in vitro* neutrophil activation system to analyze the cellular and molecular mechanisms by which the EndoS treatment reduced the pathogenicity of rabbit anti-mCOL7.

## Materials and Methods

### Rabbit Anti-mCOL7 IgG Preparation

Pathogenic rabbit anti-mCOL7 IgG was obtained from a commercial supplier (Eurogentec, Köln, Germany) and generated as previously described [10]. In brief, New Zealand white rabbits were immunized with recombinant forms of the glutathione *S*-transferase (GST)-tagged NC1 domain of mCOL7, and anti-mCOL7 IgG was purified from the rabbit serum affinity chromatography using protein G. Recombinantly expressed GST-EndoS was prepared as described [14]. The expression construct can for non for profit use be obtained at www.addgene.org/44655/. EndoS-treated rabbit anti-mCOL7 IgG was prepared as described previously [7]. Rabbit control IgG was prepared from healthy New Zealand white rabbit serum.

### Isolation of Neutrophils

Neutrophils were isolated from citrated blood of healthy donors by dextran sedimentation (Plasmasteril; Fresenius, Oberursel, Germany) followed by pancoll (PanBiotech, Aidenbach, Germany) density centrifugation [15]. More than 98% of the cells were viable as assessed by trypan blue exclusion and the percentage of neutrophils exceeded 97% in all experiments as determined by hematoxylin staining, with an amount of 1–3% remaining eosinophils as the major contaminant. Cells were suspended in CL-medium (RPMI 1640 buffered with 25 mM HEPES without phenol red; Biochrom, Berlin, Germany) before use.

### Ethics Statement

Approval for these studies was obtained from the Institutional Review board at the University of Lübeck (Lübeck, Germany; Az. 12-202A) according to the Declaration of Helsinki. All volunteers gave written informed consent.

### Evaluation of Dermal-epidermal Separation

Skin dermal-epidermal separation was evaluated using an modified *ex vivo* model [7]. Briefly, 6 µM cryosections prepared from C57BL/6J mouse tail skin were placed in the center of a Superfrost Plus microscope slide (Menzel-Gläser, Braunschweig, Germany). Skin sections were washed with PBS for 5 minutes to remove embedding medium, then incubated with 50 µl 0.2 mg/ml IgG for 60 minutes at 37°C in a humidified air incubator containing 5% CO_2_. After washing the sections with PBS twice, chambers were prepared as described and 500 µl of the neutrophil suspension (1×10^7^ cells/ml) was placed in each chamber. Incubation of neutrophils with skin sections was performed in a humidified air containing 5% CO_2_ for 3 hours at 37°C. Subsequently, chambers were disassembled, sections were washed in PBS, fixed with formalin, and subsequently stained with hematoxylin and eosin. Skin dermal-epidermal separation was evaluated by light-microscopy, and extend of dermal-epidermal separation was analyzed in a blinded fashion.

### Antibody-binding Assay

The capacity of rabbit anti-mCOL7 to bind its antigen was tested by indirect immunofluorescence (IF) staining of sections (6 µm) derived from healthy C57BL/6 mouse skin using DTAF-donkey-anti-rabbit IgG (Jackson Immunoresearch Laboratory, West Grove, PA, USA) as detection antibody. Staining intensity of immunoreactants at the DEJ was quantified with ImageJ software (http://rsbweb.nih.gov/ij/). Alternatively, binding of the antibodies to immobilized mCOL-7 (1 µg) was quantified by solid-phase ELISA using POD-goat-anti-rabbit IgG (Jackson Immunoresearch Laboratory, West Grove, PA, USA) for detection.

### Activation of Neutrophils in vitro

Activation of neutrophils *in vitro* by immobilized IC was performed as described previously with modification [13]. Briefly, mCOL7 (1 µg/ml) was coated to the bottom of a 96-well plate. After washing and blocking with PBS supplemented with 1% low-endotoxin BSA and 0.05% Tween-20, the coated mCOL7 was incubated with 100 µg/ml rabbit anti-mCOL7 IgG in PBS. After removal of unbound antibodies, generation of reactive oxygen species by neutrophils was determined by measurement of chemiluminescence in the presence of 60 µg/ml luminol (5-amino-2,3-dihydro-1,4-phthalazindione; Roche Diagnostics, Mannheim, Germany). Degranulation was determined by the amount of lactoferrin and elastase released [15]. Morphology of neutrophils was monitored by light microscopy following 1 h of stimulation.

### Immune Complex Binding Assay

Binding of immune complexes to neutrophils was tested by flow cytometry. Briefly, 5×10^5^ neutrophils were incubated at 4°C with suspended insoluble IC (prepared with 1 µg mCOL7 and 100 µg rabbit-anti-mCOL7 IgG or EndoS-treated anti-mCOL7 IgG ) for 30 min. Cell-bound IC were detected by staining with FITC-conjugated donkey-anti-rabbit IgG at 4°C.

### Statistical Analysis

Data are presented as mean ± s.d. for the number of samples indicated in the figure or figure legends. Statistically significant (P<0.05) difference among the groups were calculated using one-way analysis of variance (ANOVA) test.

## Results and Discussion

### EndoS Treatment Reduces the Pathogenicity of the Rabbit Anti-mCOL7 IgG in the ex vivo Model of EBA

**Figure 1:**
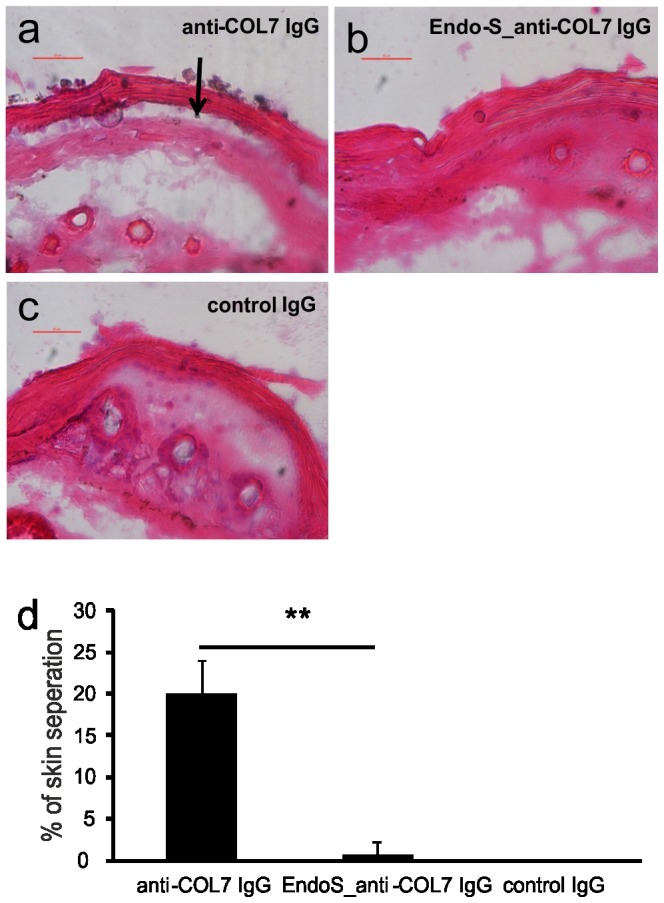
Effect of EndoS treatment of anti-mCOL7 IgG on tissue damage in an *ex vivo* model of EBA. Mouse skin cryosections were incubated with 0.2/ml rabbit-anti-mCOL7 IgG (a), or EndoS-treated 0.2 mg/ml rabbit-anti-mCOL7 IgG (b) or rabbit control IgG (c) for 1 hour at 37°C. Subsequently, specimen were exposed to freshly isolated human neutrophils. Sections of a representative experiment are shown. Arrows indicate the dermal-epidermal separation. Bar = 50 µm. Furthermore, skin separation was quantified as percentage of the length of epidermis detachment in relation to the length of the total dermal-epidermal zone (d). Data are presented as mean ± s.d. of 4 independent experiments. **indicates statistically significant differences (*p*<0.01) between EndoS-treated and untreated IgG.

In previous work we could show that EndoS treatment reduces the pathogenicity of rabbit anti-mCOL7 IgG both, in *in vivo* and in *ex vivo* models of the disease [7]. However, neither the precise deactivating principle of EndoS treatment has been elucidated so far nor could the cell population, which is functionally modulated by the modified antibodies, clearly be identified. Since the mouse skin cryosections in the *ex vivo* system were loaded with anti-mCOL7 IgG followed by administration of whole leukocytes, it is unclear whether only neutrophil functions are affected by EndoS treatment of the IgG, or whether its reduced pathogenicity also depends on the modulations of other cell types like macrophages or T cells. To optimize the *ex vivo* system, the whole leukocyte population was replaced by highly purified neutrophils and exposed to cryosections sensitized with normal rabbit anti-mCOL7 IgG or corresponding IgG pretreated with EndoS. Incubation with rabbit anti-mCOL7 IgG in this modifiied *ex vivo* system could induce the 20±4.1% dermal-epidermal separation, while EndoS-treated rabbit anti-mCOL7 IgG only induced 0.75±1.5% separation (Figure 1). Although it has previously been described that neutrophils are necessary for the induction of dermal-epidermal separation in the *ex vivo* system [12], here we can show that neutrophils are also sufficient for the induction of dermal-epidermal separation in the *ex vivo* system. Furthermore, the significant reduction in pathogenicity of the EndoS-treated antibody as compared to the untreated controls is mediated predominantly by a modulation of neutrophil functions.

### EndoS Treatment does not Alter Antigen Binding of the Rabbit Anti-mCOL7 IgG

**Figure 2:**
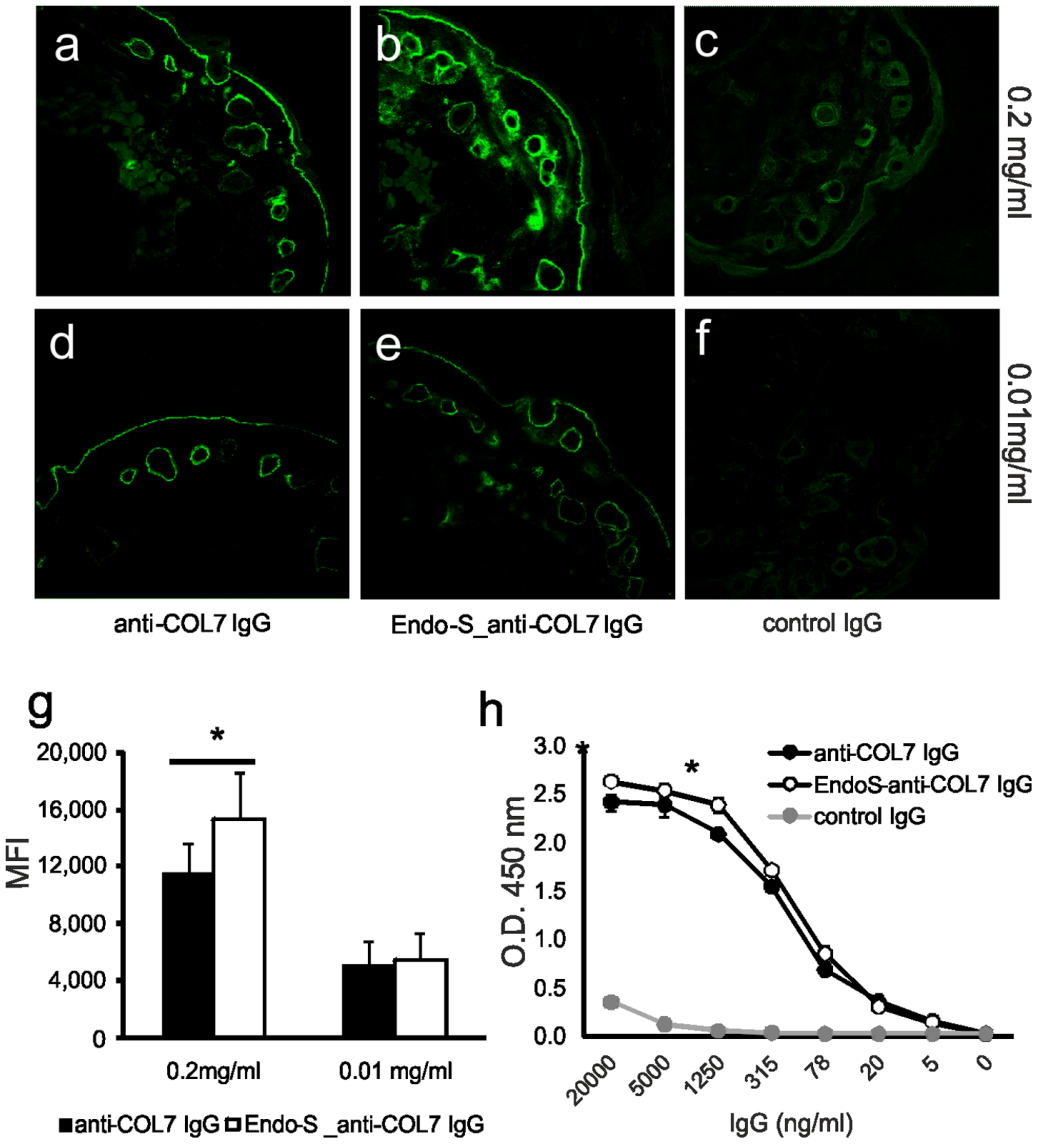
EndoS treatment of rabbit anti- mCOL7 IgG does not reduce its antigen binding capacity. Mouse skin cryosections were incubated with control rabbit IgG (a, d), anti-mCOL7 IgG (b, e) or EndoS-treated rabbit anti-mCOL7 IgG (c, f) at the concentrations indicated. Bound antibody was visualized by fluorescence microscopy using a DTAF-conjugated secondary antibody and fluorescence intensity was quantified using Image J software (g). Data of one representative experiment (a–f) are shown or given as mean ± s.d. of 8 independent experiments. Increasing concentrations of control rabbit IgG, rabbit anti-mCOL7 IgG or EndoS-treated rabbit anti-mCOL7 IgG were exposed to immobilized mCOL7 (h) and bound IgG was detected by a secondary antibody. Data are presented as mean ± s.d. n = 3, *indicates statistically significant differences (*p*<0.05) between EndoS-treated and untreated IgG.

Due to the simplicity of the *ex vivo* model system, the mechanisms how EndoS reduces the pathogenicity of the IgG can be narrowed down to two basic principles, a loss of binding of the antibody to the antigen or a decreased capacity of the immune complex to activate the neutrophils. In a first step we investigated how EndoS treatment affect the binding of the antibody to the mouse COL7 within the dermal-epidermal junction on cryosections of the skin using indirect immunofluorescence-staining. While at low concentrations of the antibodies (0.01 mg/ml) no difference in binding between EndoS-treated or untreated antibodies could be observed (Figure 2e, f, g), at high concentrations (0.2 mg/ml) the binding of EndoS-treat IgG was even slightly stronger than that of untreated IgG (Figure 2b, c, g). These results indicate that EndoS treatment does not impair antigen binding of the antibody. Moreover, our findings could be confirmed in a solid-phase ELISA with immobilized recombinant mCOL7 using ELISA. Consistent with the results in immune histology binding of EndoS-treated IgG at high concentrations (>500 ng/ml) was slightly stronger than that of untreated IgG, while this difference disappeared at lower concentrations of the antibodies (Figure 2h).

Since the EndoS-treatment only affects the Fc portion of the antibody, we hypothesize that the observed increased binding signal in samples containing EndoS-treated anti-mCOL7 IgG as compared to non-treated anti-COL7 (Figure 2h) may not be due to a difference in the antigen binding of the two antibodies but rather to a preference of the detecting secondary antibody for EndoS-treated IgG. To verify this hypothesis, we coated plastic surfaces with increasing amounts of anti-mCOL7 IgG or EndoS-treated anti-mCOL7 IgG followed by incubation with a constant concentration of the FITC-conjugated secondary antibody. No differences in binding of the second antibody to treated or untreated IgG could be detected at concentrations up to 50 ng of the coated antibodies. However, at a high dosage of the coated antibodies (500 ng), a slight but significant higher binding of the second antibody to the EndoS-treated anti-COL7 IgG than to the untreated anti-mCOL7 IgG was observed (Figure S1). Our data confirm and extend findings of a previous study *in vivo* showing that EndoS-treatment neither affects the antibody binding nor affect the complement component deposition [7].

### EndoS Treatment Affects the Capacity of Immune Complexes to Activate Neutrophils in vitro

**Figure 3:**
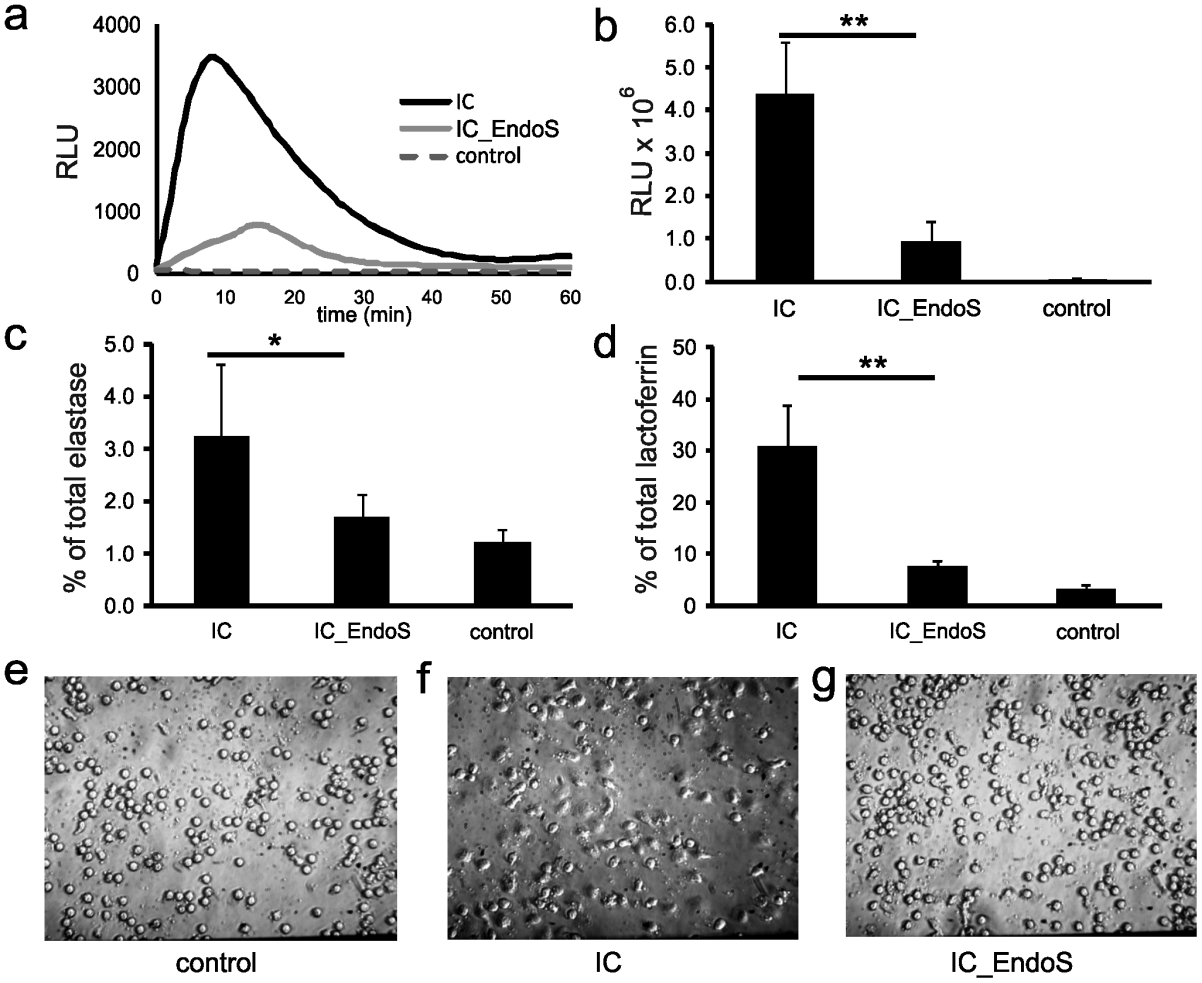
EndoS treatment of rabbit anti-mCOL7 IgG decreases neutrophil activation by immobilized immune complexes *in vitro*. Neutrophils were exposed for 1(control) or surfaces coated with IC generated from coated mCOL7 and rabbit anti-mCOL7 IgG (IC) or EndoS-treated rabbit anti-mCOL7 IgG (IC_EndoS). ROS release was quantified by chemiluminescence in the presence of luminol. Data of one representative experiment (a) are shown or given as mean ± s.d. of the integrals over one hour of 3 independent experiments (b). Neutrophil degranulation was determined by the amount of elastase (c) and lactoferrin released (d). Released proteins were determined in supernatants after 1 hour stimulation and given as percentage of their respective total amount. Data are presented as mean ± s.d. n = 3, with statically significant differences indicated by asterics (*p<0.05 and **p<0.001). Morphology of neutrophils (e–g) was analyzed by phase-contrast microscopy 1 hour after the stimulation. Data of one representative experiment out of 3are given.

In a second step we investigated whether EndoS-treatment modulates the ability of immune complexes to activate neutrophils by the use of an *in vitro* neutrophil activation model [13]. Here, neutrophils are activated by immobilized immune complex (IC) and cell activation was determined as release of reactive oxygen metabolites (ROS), degranulation, and changes in cell morphology. Neutrophils exposed to IC generated from EndoS-treated rabbit anti-mCOL7 and the corresponding antigen mCOL7 (IC_EndoS) released significant lower amounts of ROS than those activated by IC prepared from untreated rabbit anti-mCOL7 (IC) (77% reduction, Figure 3a, b). Furthermore, release of the primary granule marker elastase as well as the secondary granule marker lactoferrin was also significantly reduced in the neutrophil stimulated with IC_EndoS as compared to neutrophils treated with IC (48% reduction for elastase, Figure 3c, and 76% reduction for lactoferrin release, Figure 3d, respectively). Finally, we determined the effect of EndoS treatment on neutrophil morphology. While IC-activated neutrophils spread on the surface (Figure 3 f), IC_EndoS activated neutrophil (Figure 3g) and unstimulated neutrophils (Figure 3e) did not show any spreading. Taken together, these results clearly show that IC derived from EndoS-treated IgG have a reduced capacity to activate neutrophils. Our findings are in line with results from previous studies showing that EndoS treatment of patient derived-anti-neutrophil cytoplasmatic autoantibodies (ANCA)-drastically reduced their ability to induce neutrophil respiratory burst and degranulation [5] in a Fc-dependent manner, and that EndoS treated ICs from sytemic lupus erythematosus (SLE) patients have a reduced capacity to activate both neutrophils and plasmacytoid dendritic cells (PDCs) through FcRs [16]. Our results further substantiate the idea that the Asn-297 attached sugar moiety is essential for the function of the Fc portion in neutrophil activation by immune complexes [17].

### Decreased Binding of EndoS-treated Antibodies to Neutrophils

**Figure 4:**
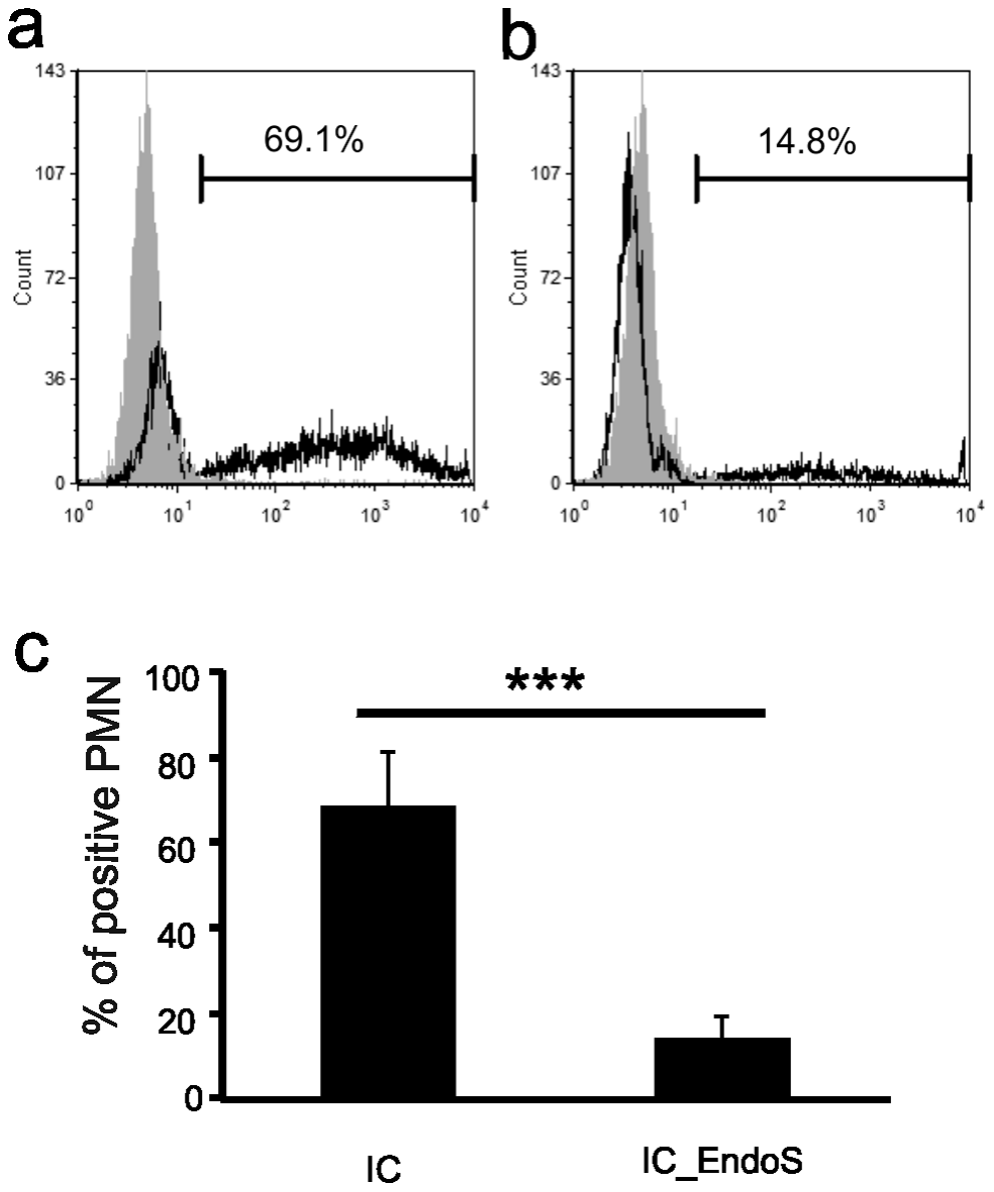
EndoS treatment of IgG decreases the binding of IC to neutrophils. Neutrophils were incubated at 4°C with suspended insoluble IC derived from untreated (a) or EndoS-treated anti-mCOL7 IgG (b) and cell-bound IC were detected by FITC-conjugated donkey-anti-rabbit IgG in flow cytometry Representative results (a, b) or mean ± s.d. of the percentage of IC-positive cells derived from 3 independent experiments (c) are shown. *indicates statistically significant differences (*p*<0.001) between EndoS-treated and untreated IgG.

The important role of IgG Fc glycans for optimal binding of the antibodies to Fcγ receptors has been described earlier [18]. Furthermore, two previous studies showed that EndoS treatment affects the interaction between IgG and FcγRs [3], [19]. Consequently, we investigated next whether the binding of IC to neutrophils is modulated after EndoS treatment. For this purpose, we first prepared insolube IC and EndoS-IC. It should be noted that we observed no differences between EndoS-treated and untreated antibodies in their capacity to precipitate soluble mCOL7 (Figure S2). Washed and suspended IC or IC_EndoS were incubated at identical concentrations with neutrophils at 4°C. Bound IC were detected by flow cytometry using fluorescence conjugated secondary antibodies against rabbit IgG. While 69.1% of the neutrophils were able to bind the untreated IC in a broad peak (Figure 4a,c), only 14.8% of the cells stained positive for IC_EndoS (Figure 4b,c). However, no binding of the secondary antibody could be detected in the absences of immune complexes. Since the FITC-conjugated secondary antibody at the concentration used binds to IC and IC_EndoS in a comparable manner (Figure S3), the decrease of the fluorescence signal on IC_EndoS-treated cells cannot be explained by a reduced binding capacity of the detecting antibody. Therefore, the dramatic reduction in the binding of IC_EndoS as compared to untreated control IC clearly indicates that deglycosylation of IgG Fc affects their binding to the FcγR on neutrophils. A recent study systemically analyzed the interaction between ICs of different human IgG subclasses (with and without the glycan at Asn-297 through mutatation or enzyme hydroysis) and the different FcRs expressed on CHO cells. This study shows that the Asn-297 is important for many, but not all, IC/FcR interactions [20]. Here we could clearly show that the Asn-297 attached sugar moiety is important for the binding of rabbit anti-mCOL7 ICs to FcγR on human neutrophils, which explains the previously shown positive *in vivo* effects of EndoS seen in the EBA model [7] as well as the inhibition of neutrophil-driven dermal-epidermal separation seen in human skin sections as presented above. In light of the previous findings that EndoS treatment of human ICs from SLE patients does not activate neutrophils or PDCs, it is likely that also human anti-COL7 ICs will be reduced in their pathogenicity by EndoS hydrolysis.

In conclusion, we could show that EndoS treatment of rabbit-anti-mCOL7 IgG reduces its pathogenicity *in vitro* and *ex vivo* by decreasing the binding of IC to FcγR on human neutrophils. This sheds new light on the mechanims of EndoS inhibition of pathogencity in the anti-COL7 EBA model and could help to explain the positive effects seen in other models. Taken together, this supports further development of IgG glycan hydrolysis, as examplified by EndoS, as a potential treatment option in antibody mediated autoimmune diseases.

## Supporting Information

Figure S1 **Binding of FITC-conjugated donkey-anti-rabbit IgG to increasing concentrations of immobilized rabbit-anti-mCOL7 IgG or EndoS-rabbit-anti-mCOL7 IgG.** Anti-mCOL7 IgG or EndoS-anti-mCOL7 IgG was coated at the concentration indicated to 96 well plates (Black IsoPlate-96 Black, PerkinElmer). After the blocking with BSA, bound IgG was detected by FITC-donkey-anti-rabbit IgG (5 µg/ml) and the fluorescence signal was recorded by a fluorescence reader (MDS Analytical technologies). *, P<0.05.
(DOCX)

Figure S2 **Precipitation of soluble mCOL7 by EndoS-treated and untreated anti-mCOL7 IgG.** Recombinant mCOL7 (3 µg) was incubated increasing amounts of control rabbit IgG, rabbit-anti-mCOL7 IgG or EndoS-rabbit-anti-mCOL7 IgG at 37 degree for 2 hours. Insoluble IC were collected after centrifugation, washed, resuspended and then quantitified photometrically at a wavelenght of λ = 280 nM.
(DOCX)

Figure S3 **Binding of increasing concentrations of FITC-conjugated donkey-anti-rabbit IgG to rabbit-anti-mCOL7 IgG and EndoS-rabbit-anti-mCOL7 IgG.** Anti-mcol7 IgG or EndoS-anti-mcol7 IgG (10 µg/ml each) was coated to 96 well plates. After the blocking, wells were incubated with FITC-donkey-anti-rabbit IgG at concentrations indicated and fluorescence was determined as described in the legend to Figure S1. *, P<0.05.
(DOCX)
