# Supplementary figures and images for: EndoS Reduces the Pathogenicity of Anti-mCOL7 IgG through Reduced Binding of Immune Complexes to Neutrophils
Source: PLoS One. 2014 Feb 4;9(2):e85317. doi: 10.1371/journal.pone.0085317 (PMC3913582; doi:10.1371/journal.pone.0085317)

**Figure S1**


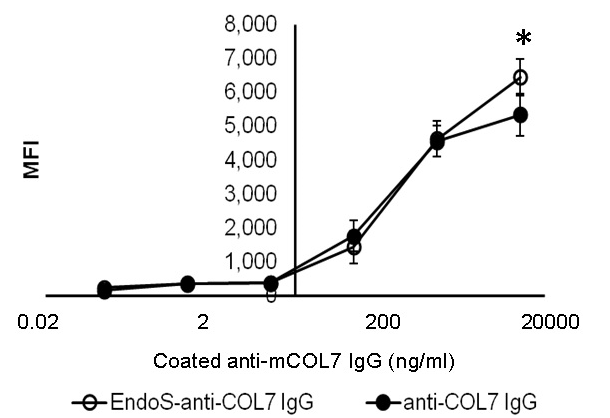

Supplement: Figure S1 — Binding of FITC-conjugated donkey-anti-rabbit IgG to increasing concentrations of immobilized rabbit-anti-mCOL7 IgG or EndoS-rabbit-anti-mCOL7 IgG. Anti-mCOL7 IgG or EndoS-anti-mCOL7 IgG was coated at the concentration indicated to 96 well plates (Black IsoPlate-96 Black, PerkinElmer). After the blocking with BSA, bound IgG was detected by FITC-donkey-anti-rabbit IgG (5 µg/ml) and the fluorescence signal was recorded by a fluorescence reader (MDS Analytical technologies). *, P<0.05. (DOCX) [file pone.0085317.s001.docx]

**Figure S3**


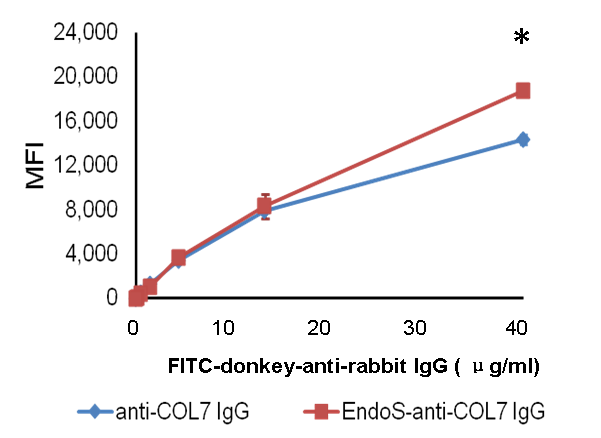

Supplement: Figure S3 — Binding of increasing concentrations of FITC-conjugated donkey-anti-rabbit IgG to rabbit-anti-mCOL7 IgG and EndoS-rabbit-anti-mCOL7 IgG. Anti-mcol7 IgG or EndoS-anti-mcol7 IgG (10 µg/ml each) was coated to 96 well plates. After the blocking, wells were incubated with FITC-donkey-anti-rabbit IgG at concentrations indicated and fluorescence was determined as described in the legend to Figure S1. *, P<0.05. (DOCX) [file pone.0085317.s003.docx]
